# Mesolimbic white matter connectivity mediates the preference for sweet food

**DOI:** 10.1038/s41598-019-40935-6

**Published:** 2019-03-13

**Authors:** Paul Francke, Lena J. Tiedemann, Mareike M. Menz, Judith Beck, Christian Büchel, Stefanie Brassen

**Affiliations:** Department of Systems Neuroscience, University Medical Centre Hamburg-Eppendorf, Martinistrasse 52, D-20246 Hamburg, Germany

## Abstract

Dopaminergic brain structures like the nucleus accumbens (NAc) are thought to encode the incentive salience of palatable foods motivating appetitive behaviour. Animal studies have identified neural networks mediating the regulation of hedonic feeding that comprise connections of the NAc with the ventral tegmental area (VTA) and the lateral hypothalamus (LH). Here, we investigated how structural connectivity of these pathways relates to individual variability in decisions on sweet food consumption in humans. We therefore combined probabilistic tractography on diffusion imaging data from 45 overnight fasted lean to overweight participants with real decisions about high and low sugar food consumption. Across all individuals, sugar preference and connectivity strength were not directly related, however, multiple regression analysis revealed interaction of mesolimbic structure and sugar preference to depend on individuals’ BMI score. In overweight individuals (BMI: ≥25 kg/m², N = 22) higher sugar preference was thereby specifically related to stronger connectivity within the VTA-NAc pathway while the opposite pattern emerged in participants with normal BMI (BMI: <25 kg/m², N = 23). Our structural results complement previous functional findings on the critical role of the human mesolimbic system for regulating hedonic eating in overweight individuals.

## Introduction

The consumption of high sugar diets is a discussed risk factor for obesity and type 2 diabetes^1^. The palatability of sugar has been linked to dopamine release in the nucleus accumbens (NAc) in rodents^2^ and there is evidence for neural adaptations in the NAc in response to excessive sugar intake^3,4^. The NAc is considered a key structure in the regulation of appetitive behaviour^5,6^. More generally, the neural control over the motivation for palatable food consumption has been predominantly assigned to dopaminergic circuits which are directly linked to homeostatic networks and metabolic signals^7–9^. Accordingly, direct stimulation and optogenetic findings in rodents have identified a metabolic-reward feeding regulation network comprising hypothalamic-mesolimbic pathways in which lateral hypothalamic (LH) signalling can activate dopaminergic midbrain structures such as the ventral tegmental area (VTA)^10^. Given strong reciprocal connections between the VTA and NAc^5^, increased activity in the VTA increases DA release into the NAc and thus affects the incentive salience of food^11^. The NAc then feeds back to inhibit LH GABAergic neurons and hereby reduces feeding drives^12^. Interestingly, while the mesolimbic pathway is well known for triggering reward-oriented behaviour^5,6^, recent studies demonstrate the NAc-LH pathway to also be specifically involved in hedonic eating regulation. In detail, optogenetic stimulation of the NAc shell^D1R^–LH^GABA^ pathway suppresses licking for a palatable reward^13^. Moreover, this connection has recently been associated with the inhibition of alcohol seeking behavior again due to D1-projections in this pathway^14^. How individual characteristics of structural connectivity within this accumbal network are related to appetitive eating behaviour in humans, however, is less understood.

Neuroimaging studies in humans^15^ and rodents^16–18^ as well as in knock-out mouse models^19^ show that experience-dependent plasticity and white matter dynamics contribute to learning and behaviour. Myelin changes through learning are partly explained by the integration of oligodendrocyte precursor cells which are capable of integrating new myelin even in adulthood^20^. Neural plasticity in mesolimbic structures has been observed in response to both drugs and natural rewards^21,22^. Thus, it could be speculated that reinforcement learning related to perpetual hedonic food consumption may trigger plastic changes in accumbal pathways, as previously suggested from data in rodents^3,4^.

Probabilistic tractography of white matter tracts allows studying structural connectivity in specific networks of the human brain. Several studies demonstrate direct associations between individual differences in behaviour and white matter connectivity (for an overview see^23^), thereby complementing functional measures as provided by functional MRI. In the context of ingestive behaviour, studies on white matter tractography have reported both decreased and increased integrity of white matter tracts in obese compared to normal weight individuals^24,25^, with some data indicating reduced connectivity in reward networks^25,26^, while others report increased white matter integrity of accumbal circuits^24^ in participants with higher BMI scores. One reason for this apparent controversy, besides genetic variability^27,28^, might be the uncontrolled impact of individual eating behaviour on brain structure. By now, however, no study has combined measures of hedonic eating behaviour with white matter integrity in reward pathways in the human brain.

In this study we aimed to elucidate the association between structural connections of the NAc–feeding regulation circuit and individual preferences to consume sweet food. Specifically, we investigated to what extent real decisions about sweet food consumption are related to individual characteristics of white matter tracts connecting the NAc with the VTA and the LH, as identified by probabilistic tractography. Since we expected an impact of current weight status on structure-function associations, our sample included both normal and overweight individuals.

## Results

### Behavioural data

**Figure 1:**
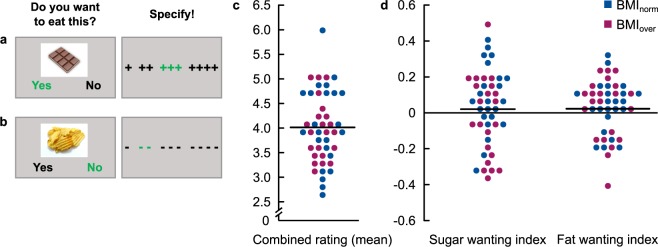
Food wanting task and behavioural data. (**a**,**b**) Food wanting task: Participants indicated in a two-step procedure how much they wanted to eat the displayed food at the moment. The wanting (**a**) or rejection (**b**) of an item was followed by 4-point rating scale ranging from weakest wanting (+) or rejection (−) to strongest wanting (++++) or rejection (− − − −). Answers selected by the participant were highlighted in green in order to provide visual feedback (**c/e**) Behavioural data: Results from the two-step ratings were combined to a 8-point food wanting score (see text for details). Mean values across items for each participant are depicted in (**c**). Within subject correlation between the item-wise combined wanting rating and sugar (fat) content are shown for each participant (see text for details) in (**d**). Every point refers to the result/data of one participant; the black line represents the mean across all participants. BMI_norm_: BMI: 18.5–24.9 kg/m², N = 23; BMI_over_: BMI ≥ 25 kg/m², BMI_over_, N = 22.

Forty-five participants were presented with a randomized series of 140 visual food stimuli, depicting food items with varying degrees of sugar content (mean sugar content: 16.48 g/100 g, SD = 19.34, range: 0–78, see Supplementary Table S1 for a list of food items). In a two-step procedure, participants were asked whether they want to eat a mouthful of the displayed food (‘Yes’ versus ‘No’). The decision was followed by a detailed four-point rating indicating how much they want (‘+’ = weakest wanting, ‘++++’ = strongest wanting) or refuse (‘−’ = weakest rejection,‘− − − −’ = strongest rejection) to consume each food (Fig. 1a,b). These two steps were combined to a single wanting value per item, ranging from 1 (first response = ‘no’, second response = ‘− − − −’), the strongest rejection, to 8 (first response = ‘yes’, second response = ‘++++’) the strongest wanting to consume the food. Importantly, participants were informed that they would be offered and have to eat a mouthful of one randomly selected preferred food item after the experiment. To address our research questions, food items were analyzed with respect to sugar and fat content (control condition). As a marker of sugar (fat) wanting, within-subject Pearson correlations were computed between the trial-wise 8-point combined rating score and the sugar (fat) content of each item: i.e. sugar wanting index = corr(g sugar/100 g, combined wanting score). Hereby, higher correlations can be considered as a stronger willingness to consume food with high sugar (fat) content.

On average, participants wanted to consume (“yes”-decision) 41.17% (SD = 16.91) of the 140 food items. The mean parametric willingness to consume the food on the 8-point combined rating scale was 4.02 (SD = 0.73, Fig. 1c). Neither the sugar wanting index (mean r = 0.02, SD = 0.21) nor the mean fat wanting index (mean r = 0.02, SD = 0.16) differed from zero (all *p* > 0.32, Fig. 1d), i.e. across all participants there was no significant effect of sugar or fat content on food preferences. As obvious in Fig. 1d, however, there was considerable variability in individual correlation coefficients.

Across all participants, none of these behavioural measures was directly related to individual BMI scores (all *p* > 0.21) nor differed between groups with normal (BMI < 25 kg/m², N = 23) and overweight BMI (BMI ≥ 25 kg/m², N = 22) (all *p* > 0.16).

### White matter connectivity strength and interactions with behaviour

**Figure 2:**
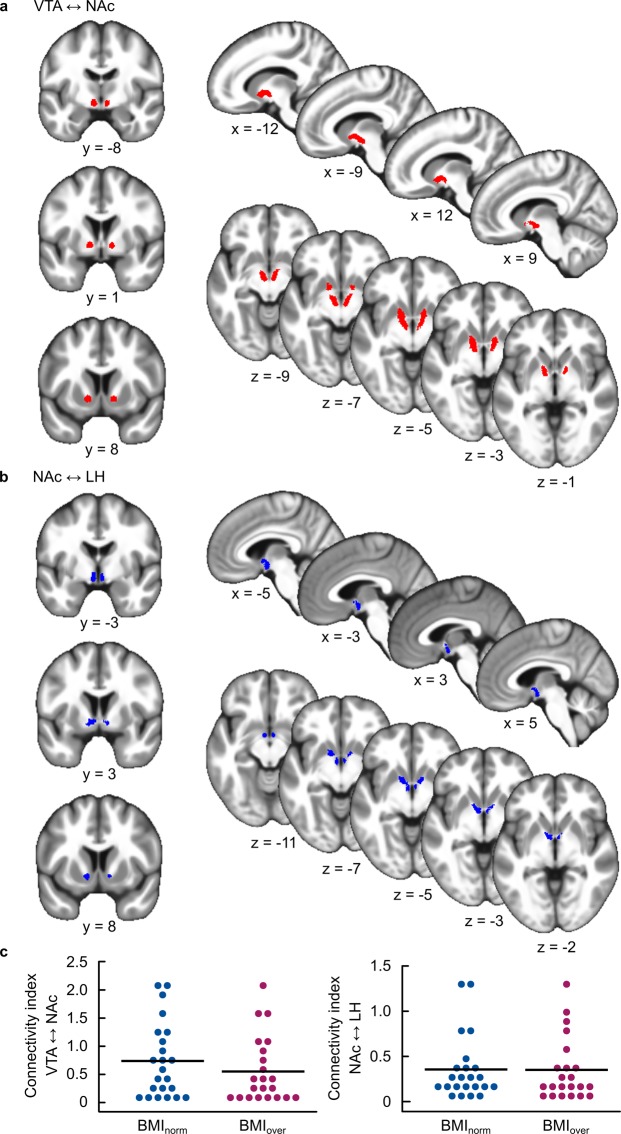
Results from structural connectivity analyses. (**a**,**b**) Group probability maps of VTA-Nac (**a**) and NAc-LH (**b**) tracts. Individual participant’s probabilistic tractography results were transformed into standard space, binarized, and summed across all subjects. For illustration purposes, the summed tract images were thresholded to show only overlapping pathways in 75% of subjects (i.e. N = 34). (**c**) Single-subject connectivity indices separated by BMI group. Every point refers to the data of one participant; the black line represents the mean across all participants. BMI_norm_: BMI: 18.5–24.9 kg/m², N = 23; BMI_over_: BMI ≥ 25 kg/m², N = 22.

A regions of interest (ROI) based approach was used to analyse the strength of structural connections between the NAc and the VTA as well as the NAc and the LH. Tracking was performed in both directions for each pathway and resulting streamlines were summed to obtain a single connectivity measure per pathway. Since we had no hypotheses on lateralization, data were collapsed across hemispheres. Connection probability was then defined as mean number of streamlines per voxel sampled (= sum of streamlines across all voxels from a specific tract divided by the number of voxels in the individual seed and target masks of this tract^29^; see Methods for further details). Group tractography results of both tracts are illustrated in Fig. 2a,b. The connectivity index of neither VTA-Nac nor NAc-LH was directly correlated with BMI (all *p* > 0.64), nor was there any difference between BMI groups (all *p* > 0.33, see Fig. 2c). There was also no direct correlation between connectivity strength and sugar or fat wanting (all *p* > 0.21). See Supplementary Results for further details on tract differences.

In the next step, we tested more complex brain-behaviour interactions with respect to weight status^30,31^, i.e. we tested whether white matter connectivity strength in accumbal pathways is differentially related to hedonic eating behaviour in participants with higher or lower BMI scores. To this end, we built two multiple regression models in which individual within-subject correlation indices of sugar wanting (i.e. sugar wanting index) served as the dependent variable while either (Reg. 1) mean centered VTA-NAc connectivity index, BMI, as well as the BMI interactions with VTA-NAc (BMI x VTA-NAc) or (Reg. 2) mean centered NAc-LH connectivity index, BMI as well as BMI interactions with NAc-LH (BMI x NAc-LH) were entered as independent predictors.

Results showed that the regression model Reg. 1 including VTA-NAc was highly significant, i.e. explained significant variability in sugar wanting (R^2^ = 0.34, F(3,41) = 7.03, *p* = 0.001). This was driven by a significant predictive value of the interaction between BMI and VTA-NAc connectivity strength (β = 0.56, *p* < 0.001; Supplementary Table S2). In contrast, the regression model Reg. 2 including NAc-LH revealed no significant results (R^2^ = 0.04, F(3,41) = 1.97, *p* = 0.13). The specificity of the VTA-NAc result was further tested in a third regression model including both tracts, BMI and their interactions. Underlining the results from Reg. 1, the model explained significant variability in individuals’ sugar wanting index (R = 0.38, F(5,39) = 4.79, *p* = 0.002) again uniquely by BMI interaction with VTA-NAc connectivity (β = 0.62, *p* < 0.001). The control analyses including fat wanting (Reg. 1–3), in contrast, revealed no significant results (see Supplementary Table S2).

**Figure 3:**
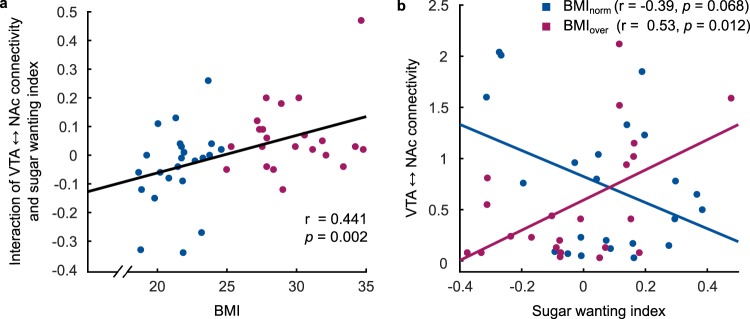
Correlation between VTA-NAc structure and sugar wanting and its relationship with BMI. (**a**) The interaction of sugar wanting index and VTA-NAc connectivity correlated significantly with BMI. (**b**) Further exploration of this effect shows a significantly positive correlation between sugar wanting index and VTA-NAc connectivity only in the BMI_over_ group, which differed significantly from the correlation within the BMI_norm_ group (Z = 3.13, *p* = 0.001). Plots of non-significant results for the VTA-NAc tract are provided in the Supplementary Fig. S1.

In order to further illuminate the significant impact of the interaction between BMI and VTA-NAc connectivity on sugar wanting, we directly tested for BMI effects on structure-function interactions. Results revealed a significantly positive correlation (r = 0.44, *p* = 0.002), i.e. individuals with higher BMI scores demonstrated a stronger positive interaction between sugar preference and VTA-NAc connectivity (Fig. 3a). To finally investigate the nature of structure-function interaction in different BMI groups, we tested the correlation between VTA-NAc connectivity and sugar wanting index separately for participants with normal BMI scores (BMI_norm_) and overweight participants (BMI_over_). Results demonstrate a significantly positive correlation between sugar wanting and VTA-NAc connectivity in overweight subjects (r = 0.53, *p* = 0.012) and a trend towards significance for a negative structure-function interaction in the normal weight group (r = −0.39, *p* = 0.068) (Fig. 3b). Consequently, correlations differed significantly between groups (Z = 3.13, *p* = 0.001).

## Discussion

Our findings reveal distinct patterns of associations between white matter brain structure and sugar preference in normal- and overweight participants. In fasted overweight individuals, estimated connections between the NAc and the VTA predicted the motivation to consume sweet food. The stronger the white matter connectivity within this mesolimbic pathway, the more individuals’ food wanting was driven by the sugar content of foods. Importantly, this finding was restricted to overweight individuals while in normal weight participants, we observed a trend towards an opposite association, i.e. mesolimbic connectivity was related to less sugar wanting. Findings were restricted to the VTA-NAc pathway which has recently been related to motivational behaviour including palatable food seeking and consumption in rodents^12,32–34^ and the regulation of hedonic food choices^7–9,11^. Previous studies found that sucrose licking leads to increased levels of dopamine in the NAc of rats^2,35^ and that binge-like sucrose consumption results in an increase in extracellular dopamine in the NAc^3,36^. Especially the NAc-shell has thereby been found to encode the palatability and reward of sucrose^35,37^. In humans, the intake of high sweet soft drinks and milkshake activates the striatum including the NAc^38^ and VTA activation has been related to chocolate consumption^39^. Both the NAc as well as the VTA have been shown to demonstrate neuroplastic changes in response to drug and natural reward consumption^21,22^. Moreover, recent rodent data show increased spine densities at the distal branch orders of the NAc shell neurons following a 12-week long-term binge-like sucrose consumption^4^. The NAc shell receives inputs not only from dopaminergic afferents that project from the VTA^12,40^ but also from glutamatergic afferents from regions engaged during reinforcement learning like the basolateral amygdala and the hippocampus^41^. Thus, our findings may indicate that perpetual sugar consumption in the past in our overweight participants with higher sugar preference may have triggered neuroplastic white matter changes. Due to the cross-sectional nature of our study, we cannot rule out that our findings may rather represent a cause than a consequence of hedonic eating behaviour. Interestingly and in contrast to findings in overweight participants, in our normal weight sample we observed a trend towards lower mesolimbic connectivity to be associated with higher sugar preference. There is now evidence that frequently observed grey matter changes in overweight and obesity overlap with genetic vulnerability factors. It has been speculated that genetic effects on brain morphology and cognitive function, e.g. reduced prefrontal self-regulation, may promote hedonic overeating resulting in overweight and obesity^42^. Moreover, one recent study shows reduced NAc-thalamus white matter connectivity in non-obese carriers of obesity-risk alleles of the fat mass and obesity associated (FTO) gene indicating disruptions in mesolimbic white matter microstructure to be a potential, early predisposing factor for the development of obesity^28^. Thus, one could speculate that our findings indicate differential functional roles of white matter mesolimbic connectivity in individuals with and without overweight. While in normal weight individuals, lower connectivity may represent a (genetic) risk or vulnerability factor for hedonic overeating and obesity in the future^9,43^, perpetual hedonic overeating in the past in now overweight and obese individuals may have triggered neuroplastic changes in the mesolimbic system.

Of course, this speculative hypothesis needs to be tested in longitudinal studies on larger data sets. In any case, our findings complement functional data on the critical role of mesolimbic brain structures in regulating hedonic eating behaviour which may be aberrant in overweight^9,11,12,32,44^.

We did not observe overall altered sugar preference or VTA-NAc connectivity strength in our overweight sample. The few existing DTI tractography studies in the field of ingestive behaviour have produced mixed findings regarding the wiring within reward networks. While one study reported greater connectivity from regions of the reward network to cortical areas in an overweight compared to a normal weight group^24^, others found lower numbers of streamlines between the NAc and the rest of the reward network in obese individuals^26^. Inconsistent findings have previously also been reported for other DTI measures such as fractional anisotropy (FA), and been discussed as a potential consequence of competing metabolic processes affecting white matter integrity in overweight and obesity such as insulin resistance, inflammation and dyslipidaemia (for an overview see^45^. Given that most of our overweight participants were non-obese (14 out of 22) these factors might be less relevant here, but however cannot be ruled out due to sample size limitations. In addition, it may be possible that with increasing overweight, compensatory behavioural strategies like restrained eating may have diminished direct associations of weight with eating behaviour. Lower sugar wanting values in some overweight individuals may thereby result from the engagement of learned higher order cognitive strategies such as self-control and restrained eating accomplished to manage weight^46–48^. In this context, it would be interesting for future studies to include white matter mesocortico-limbic pathways and to combine these structural data with functional paradigms that are sensitive to self-regulation (e.g. by attentional manipulation of health considerations^48^).

Structure-function associations in this study were restricted to the mesolimbic pathway, while no significant interactions were found for the tract between the NAc and the LH. Very recent findings have indicated a critical role of this pathway as a negative regulator of feeding and reward^13,14,49^, even though, in contrast to the mesolimbic system, so far findings are restricted to rodents. It could be speculated that our behavioral paradigm was not suited to assess functions thought to be encoded by this pathway. For instance, tasks involving food (over-)consumption (e.g. ad-libitum eating task) and/or designs where participants’ ability to regulate hedonic food intake can be assessed may be more appropriate to study structure-function associations in this context. In any case, our differential results further strengthen the specificity of findings on the role of the mesolimbic pathway for sugar preference as assessed in this study.

Finally, variability and weight-dependence in sugar preference might be reduced in the present study due to the fact that all participants were investigated after an overnight fast. This was done to rule out systematic effects of physiological state and food craving on neurobehavioral findings, on the other hand it may have lowered the effect of individual eating habits on food preferences^50^. Future studies in larger samples may be able to investigate the impact of potential modulating variables such as cognitive control, physical activity and metabolic factors.

White matter tractography is still the central computational reconstruction method when studying connectivity in the human brain non-invasively. Compared to a high sensitivity of this method as indicated by a strong overlap of reconstructed tracts with ground truth bundles, the specificity has been questioned given the high amount of observed invalid bundles within tractograms^51^. This should be considered when interpreting our results, even though our specific findings of structure-function associations depending on weight status when including the VTA-NAc but not the NAc-LH pathway and when including sugar but not fat wanting argue for a certain specificity of our results.

In conclusion, our findings provide first evidence that individual differences in the motivation to consume sweet food is related to the structural connectivity within mesolimbic pathways and that this structure-function associations differs depending on current weight status. Mesolimbic wiring may thereby represent either a potential cause or consequence of hedonically driven food consumption which can promote overweight and obesity.

## Materials and Methods

### Participants and study protocol

Data for this study were collected within a multimodal collaborative research project on ingestive behaviour and neural networks, in which participants from different weight and age groups were investigated using behavioural and neural measures. All younger individuals (age <35 years) in whom DTI data were acquired were included in the present study. General exclusion criteria comprised current or previous psychiatric or neurological disorders, chronic and acute physical illness including diabetes, current psychopharmacological medication as well as MR-specific exclusion criteria. No participant followed any specific diet at the time of the experiment. To exclude systematic confounds during food evaluation, severe food allergies and adherence to a vegan diet constituted further exclusion criteria. The study was conducted in accordance with the ethical principles of the Declaration of Helsinki and was approved by the Ethics Committee of the Medical Council of Hamburg. All participants gave written informed consent and were financially compensated for participation.

Forty-eight participants fulfilled the inclusion criteria. Three out of those, however, had to be excluded due to imaging artefacts which resulted in a final sample of 45 participants (22 women; mean age 25.80 years, SD = 3.19; mean BMI: 25.50 kg/m², SD = 4.73). Participants were sampled into groups with normal BMI scores (BMI: 18.5–24.9 kg/m², BMI_norm_, N = 23) or overweight BMI scores (BMI ≥ 25 kg/m², BMI_over_, N = 22). Groups did not differ with respect to age, sex, fasting glucose levels, fasting time and reported hunger (all *p* > 0.10; s. Supplementary Table 2). Hunger ratings were assessed immediately before the behavioural experiment started by using a ten-point rating scale from 0 (= not hungry at all) to 10 (= very hungry).

For the neural and behavioural measures reported in this study, participants were asked to arrive in the laboratory between 7.45 and 9.45 a.m. after an overnight fast of at least ten hours (mean fasting time: 12.99 hours, SD = 1.42). Fasting glucose levels confirmed fasting state in all participants (mean blood glucose: 4.83 mmol/l, SD = 0.38). Normal HbA1C values confirmed the absence of diabetes in our sample (mean HbA1C: 4.95%, SD = 0.30).

### Decision task about food consumption

During the experiment, participants were presented with 140 visual stimuli depicting food items (for a list of food items see Supplementary Table S1). All pictures had a size of 400 × 400 pixels and were presented on a white background. Please note that brain activity was measured during the behavioural task but is part of another project and will be published elsewhere. First, participants answered a binary question regarding whether they want to eat a mouthful of the displayed food at that very moment. Next, participants specified their respective decision on scales from one to four, indicating how strongly they want or refuse to consume each food (Fig. 1a,b). Importantly, participants were informed that they would be offered and have to eat a mouthful of one randomly selected preferred food item after the experiment.

### Diffusion tensor imaging

Data were acquired on a 3 T whole-body MR system (TIM Trio, Siemens Healthcare, Erlangen, Germany) using a 32-channel head coil. Diffusion imaging was performed using simultaneous multi-slice acquisition (acceleration factor 2) with the blipped-CAIPI approach^52^. Each volume comprised 86 slices (thickness 1.5 mm, no gap) covering the whole brain with a FOV of 218 × 218 mm and a base resolution of 144 resulting in a voxel size of 1.5 mm^3^. 7/8 partial Fourier imaging was used. Sixty directions derived from a buckyball were acquired with a diffusion weighting of b = 1000 s/mm² interleaved with six images without diffusion weighting (b = 0 s mm^2^) yielding a TE of 110 ms and a TR of 6600 ms. This acquisition was supplemented by two additional non-diffusion weighted images with inverted phase encoding direction. Additionally, an MPRAGE structural image (240 slices, voxel size 1 × 1 × 1 mm) was acquired for co-registration and anatomical overlay.

### Data analysis

#### Behavioural data

Behavioural data were analysed with Matlab R2016 (MathWorks) and SPSS Statistics 22 (IBM). To address our research questions, food items were analysed with respect to sugar and fat content (control condition). As a marker of sugar (fat) wanting, within-subject correlations were computed between the trial-wise 8-point rating and the sugar (fat) content of each item (see Results for details). Hereby, high correlations can be considered as a stronger willingness to consume food with high sugar (fat) content.

#### Diffusion imaging data

Diffusion data were preprocessed and analysed using FSL version 5.09 (https://www.fmrib.ox.ac.uk/fsl). To improve image quality, reference volumes with reversed phase-encoded blips were used to estimate and correct for the susceptibility-induced off-resonance field using TOPUP^53,54^. Diffusion weighted images of each participant were corrected for head motion and eddy currents by registering diffusion images to the b = 0 s/mm² images^55^. Additionally, the B-matrix, which contains the diffusion gradient orientations, has been updated after applying head motion correction to improve fibre tracking^56^. Next, anatomical connectivity for each subject was estimated using the FMRIB’s Diffusion Toolbox (FDT; http://www.fmrib.ox.ac.uk/fsl/fdt/index.html). The software module BEDPOSTX then allowed to infer a local model of fibre bundles orientations in each voxel of the brain. Up to two crossing fibre bundles were estimated in each voxel based on the b-values and the resolution of our data^57,58^. Compared with single-fibre models, more reliable results can be expected from dual fibre models which can account for crossing fibres. Probabilistic fibre tractography was then computed in each subject’s individual space, using the software tool PROBTRACKX2 with seed and target masks and estimating the most likely location and strength of the fibre pathway between the two areas^57–59^. The connection probability is represented by the number of tracts that reach a target voxel from a given seed. The parameters used for tractography were 5000 samples per seed voxel with a curvature threshold of 0.1, a step length of 0.5 and a maximum number of 2000 steps. Although measures derived from tractography do not directly represent fibre anatomy, they provide a valid measure of connectivity between areas^60^.

Based on previous findings (e.g.^11^) masks were created centred on peak voxels that were derived from meta-analyses conducted on the neurosynth.org platform in MNI-1 mm for the estimation of tract strength between VTA-NAc as well as NAc-LH pathways (LH: ±4 -4 -12; 2 mm radius; VTA: ±4 -14 -12; 4 mm radius; NAc ±12 10 -8; 4 mm radius, status July 2017). Tracking was performed in each individual’s native space to which each mask was registered by a combination of linear and non-linear deformation fields. Visual inspection ensured that the location of masks was anatomically plausible. Masks served as both seeds and targets (see below).

The connectivity strength is approximated by the number of tracts from each seed that reached the target^23,61,62^. Tracking was performed in both directions for each pair of seed and target region and resulting connectivity measures were summed to obtain a single connectivity measure per pathway^62,63^. Since there were no hypotheses on lateralization, the data of each pathway were collapsed across the hemispheres^64^. To account for a potential bias toward nearby connections, tractography results were adjusted by the length of the pathway as implemented in FSL. Finally, a connectivity index was calculated that reflects the mean connection probability of each voxel in the seed/targets of the respective tract. Specifically, the mean number of streamlines sampled per voxel was calculated by dividing the sum of streamlines across all voxels from a specific tract by the number of voxels in the individual seed and target masks of this tract (e.g. sum of streamlines for VTA ↔ NAc/number of voxels in individual VTA and NAc ROIs).

To assess the predictive value of NAc connections with the VTA and the LH for participants’ sugar wanting, VTA-NAc and NAc-LH connectivity indices were entered as potential exploratory variables into multiple regression models for the prediction of individuals’ sugar wanting. To evaluate the specificity of potential results on sugar wanting, we repeated our analyses using the fat content of the items as a control.

## Supplementary information

Supplementary information

## Data Availability

Behavioural data will be made available on figshare.com. Imaging data will be made available on neurovault.org. Custom code will be made available in a community repository (e.g. GitHub).

## References

[1] Luger M (2017). Sugar-Sweetened Beverages and Weight Gain in Children and Adults: A Systematic Review from 2013 to 2015 and a Comparison with Previous. Studies. Obes. Facts.

[2] Hajnal A, Smith GP, Norgren R (2004). Oral sucrose stimulation increases accumbens dopamine in the rat. Am. J. Physiol. Regul. Integr. Comp. Physiol..

[3] Avena NM, Rada P, Hoebel BG (2008). Evidence for sugar addiction: Behavioral and neurochemical effects of intermittent, excessive sugar intake. Neurosci. Biobehav. Rev..

[4] Klenowski PM (2016). Prolonged Consumption of Sucrose in a Binge-Like Manner, Alters the Morphology of Medium Spiny Neurons in the Nucleus Accumbens Shell. Front. Behav. Neurosci..

[5] Haber SN, Knutson B (2009). The Reward Circuit: Linking Primate Anatomy and Human Imaging. Neuropsychopharmacology.

[6] Sescousse G, Caldú X, Segura B, Dreher J-C (2013). Processing of primary and secondary rewards: a quantitative meta-analysis and review of human functional neuroimaging studies. Neurosci. Biobehav. Rev..

[7] Berridge KC (2009). ‘Liking’ and ‘wanting’ food rewards: brain substrates and roles in eating disorders. Physiol. Behav..

[8] Berthoud H-R (2011). Metabolic and hedonic drives in the neural control of appetite: who is the boss?. Curr. Opin. Neurobiol..

[9] Volkow ND, Wang G-J, Baler RD (2011). Reward, dopamine and the control of food intake: implications for obesity. Trends Cogn. Sci..

[10] Wise RA (1978). Catecholamine theories of reward: a critical review. Brain Res..

[11] Tiedemann LJ (2017). Central insulin modulates food valuation via mesolimbic pathways. Nat. Commun..

[12] Stuber GD, Wise RA (2016). Lateral hypothalamic circuits for feeding and reward. Nat. Neurosci..

[13] O’Connor EC (2015). Accumbal D1R Neurons Projecting to Lateral Hypothalamus Authorize Feeding. Neuron.

[14] Gibson GD (2018). Distinct Accumbens Shell Output Pathways Promote versus Prevent Relapse to Alcohol Seeking. Neuron.

[15] Scholz J, Klein MC, Behrens TEJ, Johansen-Berg H (2009). Training induces changes in white-matter architecture. Nat. Neurosci..

[16] Blumenfeld-Katzir T, Pasternak O, Dagan M, Assaf Y (2011). Diffusion MRI of Structural Brain Plasticity Induced by a Learning and Memory Task. PLOS ONE.

[17] Sampaio-Baptista C, Johansen-Berg H (2017). White Matter Plasticity in the Adult Brain. Neuron.

[18] Sampaio-Baptista C (2013). Motor Skill Learning Induces Changes in White Matter Microstructure and Myelination. J. Neurosci..

[19] McKenzie IA (2014). Motor skill learning requires active central myelination. Science.

[20] Swire M, Ffrench-Constant C (2018). Seeing Is Believing: Myelin Dynamics in the Adult CNS. Neuron.

[21] Pitchers KK (2013). Natural and Drug Rewards Act on Common Neural Plasticity Mechanisms with ΔFosB as a Key Mediator. J. Neurosci..

[22] Pitchers KK (2014). Endogenous Opioid-Induced Neuroplasticity of Dopaminergic Neurons in the Ventral Tegmental Area Influences Natural and Opiate Reward. J. Neurosci..

[23] Forstmann BU (2010). Cortico-striatal connections predict control over speed and accuracy in perceptual decision making. Proc. Natl. Acad. Sci..

[24] Gupta A (2015). Patterns of brain structural connectivity differentiate normal weight from overweight subjects. NeuroImage Clin..

[25] Riederer, J. W., Shott, M. E., Deguzman, M., Pryor, T. L. & Frank, G. K. W. Understanding Neuronal Architecture in Obesity through Analysis of White Matter Connection Strength. *Front. Hum. Neurosci*. **10** (2016). 10.3389/fnhum.2016.00271 PMC4893484 27375463

[26] Marqués-Iturria I (2015). Affected connectivity organization of the reward system structure in obesity. NeuroImage.

[27] García-García, I. *et al*. Neuroanatomical differences in obesity: meta-analytic findings and their validation in an independent dataset. *Int. J. Obes. 2005*, 10.1038/s41366-018-0164-4 (2018). 10.1038/s41366-018-0164-4 30022057

[28] Olivo G, Latini F, Wiemerslage L, Larsson E-M, Schiöth HB (2018). Disruption of Accumbens and Thalamic White Matter Connectivity Revealed by Diffusion Tensor Tractography in Young Men with Genetic Risk for Obesity. Front. Hum. Neurosci..

[29] Theisen F (2017). Evaluation of striatonigral connectivity using probabilistic tractography in Parkinson’s disease. NeuroImage Clin..

[30] Dietrich A, Hollmann M, Mathar D, Villringer A, Horstmann A (2016). Brain regulation of food craving: relationships with weight status and eating behavior. Int. J. Obes. 2005.

[31] Ribeiro, G. *et al*. Association between hedonic hunger and body-mass index versus obesity status. *Sci. Rep*. **8** (2018). 10.1038/s41598-018-23988-x PMC5895788 29643337

[32] Berridge KC, Kringelbach ML (2015). Pleasure Systems in the Brain. Neuron.

[33] Labouèbe G (2013). Insulin induces long-term depression of ventral tegmental area dopamine neurons via endocannabinoids. Nat. Neurosci..

[34] Nieh, E. H. *et al*. Inhibitory Input from the Lateral Hypothalamus to the Ventral Tegmental Area Disinhibits Dopamine Neurons and Promotes Behavioral Activation. *Neuron* 1–13, 10.1016/j.neuron.2016.04.035 (2016). 10.1016/j.neuron.2016.04.035 PMC4961212 27238864

[35] Taha SA, Fields HL (2005). Encoding of palatability and appetitive behaviors by distinct neuronal populations in the nucleus accumbens. J. Neurosci. Off. J. Soc. Neurosci..

[36] Rada P, Avena NM, Hoebel BG (2005). Daily bingeing on sugar repeatedly releases dopamine in the accumbens shell. Neuroscience.

[37] Villavicencio, M., Moreno, M. G., Simon, S. A. & Gutierrez, R. Encoding of Sucrose’s Palatability in the Nucleus Accumbens Shell and Its Modulation by Exteroceptive Auditory Cues. *Front. Neurosci*. **12** (2018). 10.3389/fnins.2018.00265 PMC5945833 29780300

[38] Burger KS, Stice E (2014). Neural responsivity during soft drink intake, anticipation, and advertisement exposure in habitually consuming youth. *Obes*. Silver Spring Md.

[39] Small DM, Zatorre RJ, Dagher A, Evans AC, Jones-Gotman M (2001). Changes in brain activity related to eating chocolate: from pleasure to aversion. Brain J. Neurol..

[40] Beier KT (2015). Circuit Architecture of VTA Dopamine Neurons Revealed by Systematic Input–Output Mapping. Cell.

[41] Brog JS, Salyapongse A, Deutch AY, Zahm DS (1993). The patterns of afferent innervation of the core and shell in the ‘accumbens’ part of the rat ventral striatum: immunohistochemical detection of retrogradely transported fluoro-gold. J. Comp. Neurol..

[42] Vainik U (2018). Neurobehavioral correlates of obesity are largely heritable. Proc. Natl. Acad. Sci. USA.

[43] Stice, E. & Yokum, S. Neural Vulnerability Factors That Increase Risk for Future Weight Gain. *Psychol. Bull*. No Pagination Specified, 10.1037/bul0000044 (2016). 10.1037/bul0000044 PMC4824640 26854866

[44] Kenny PJ (2011). Reward Mechanisms in Obesity: New Insights and Future Directions. Neuron.

[45] Kullmann S, Schweizer F, Veit R, Fritsche A, Preissl H (2015). Compromised white matter integrity in obesity: Obesity and the brain. Obes. Rev..

[46] Hare TA, Camerer CF, Rangel A (2009). Self-Control in Decision-Making Involves Modulation of the vmPFC Valuation System. Science.

[47] Kahathuduwa CN, Boyd LA, Davis T, O’Boyle M, Binks M (2016). Brain regions involved in ingestive behavior and related psychological constructs in people undergoing calorie restriction. Appetite.

[48] Hare TA, Malmaud J, Rangel A (2011). Focusing attention on the health aspects of foods changes value signals in vmPFC and improves dietary choice. J. Neurosci. Off. J. Soc. Neurosci..

[49] Rossi MA, Stuber GD (2018). Overlapping Brain Circuits for Homeostatic and Hedonic Feeding. Cell Metab..

[50] Epstein LH, Truesdale R, Wojcik A, Paluch RA, Raynor HA (2003). Effects of deprivation on hedonics and reinforcing value of food. Physiol. Behav..

[51] Maier-Hein KH (2017). The challenge of mapping the human connectome based on diffusion tractography. Nat. Commun..

[52] Setsompop K (2012). Blipped-controlled aliasing in parallel imaging for simultaneous multislice echo planar imaging with reduced g-factor penalty. Magn. Reson. Med..

[53] Andersson JLR, Skare S, Ashburner J (2003). How to correct susceptibility distortions in spin-echo echo-planar images: application to diffusion tensor imaging. NeuroImage.

[54] Smith SM (2004). Advances in functional and structural MR image analysis and implementation as FSL. NeuroImage.

[55] Andersson JLR, Sotiropoulos SN (2016). An integrated approach to correction for off-resonance effects and subject movement in diffusion MR imaging. NeuroImage.

[56] Leemans A, Jones DK (2009). The B-matrix must be rotated when correcting for subject motion in DTI data. Magn. Reson. Med..

[57] Behrens TEJ (2003). Characterization and propagation of uncertainty in diffusion-weighted MR imaging. Magn. Reson. Med..

[58] Behrens TEJ, Berg HJ, Jbabdi S, Rushworth MFS, Woolrich MW (2007). Probabilistic diffusion tractography with multiple fibre orientations: What can we gain?. NeuroImage.

[59] Johansen-Berg, H. & Behrens, T. E. J. *Diffusion MRI: From Quantitative Measurement to In vivo Neuroanatomy*. (Academic Press, 2013).

[60] Dyrby TB (2007). Validation of *in vitro* probabilistic tractography. NeuroImage.

[61] Eickhoff SB (2010). Anatomical and Functional Connectivity of Cytoarchitectonic Areas within the Human Parietal Operculum. J. Neurosci..

[62] Blank H, Anwander A, von Kriegstein K (2011). Direct Structural Connections between Voice- and Face-Recognition Areas. J. Neurosci..

[63] Bridge H, Thomas O, Jbabdi S, Cowey A (2008). Changes in connectivity after visual cortical brain damage underlie altered visual function. Brain.

[64] Cohen MX (2011). Hippocampal-Prefrontal Connectivity Predicts Midfrontal Oscillations and Long-Term Memory Performance. Curr. Biol..

