# Supplementary material for: Mesolimbic white matter connectivity mediates the preference for sweet food
Source: Sci Rep. 2019 Mar 13;9:4349. doi: 10.1038/s41598-019-40935-6 (PMC6416305; doi:10.1038/s41598-019-40935-6)
Supplement: Supplementary file 1 — Supplementary information [file 41598_2019_40935_MOESM1_ESM.docx]

**Supplementary information**

***Mesolimbic white matter connectivity mediates the preference for sweet food***

**Authors and affiliations**

Paul Francke ^1^, Lena J. Tiedemann ^1^, Mareike M. Menz ^1^, Judith Beck ^1^, Christian Büchel ^1^, Stefanie Brassen ^1*^

^1^ Department of Systems Neuroscience, University Medical Centre Hamburg-Eppendorf, Martinistrasse 52, D-20246, Hamburg, Germany

* Correspondence should be addressed to Stefanie Brassen, Department of Systems Neuroscience, University Medical Centre Hamburg-Eppendorf, Martinistrasse 52, D-20246, Hamburg, Germany. Email: [sbrassen@uke.de](mailto:sbrassen@uke.de)

**Supplementary Table S1: Food items and sugar/fat content**

|  |  | |  |  |  | | | |  |  |
| --- | --- | --- | --- | --- | --- | --- | --- | --- | --- | --- |
| **Food (total=140)** | **Sugar/fat content (g/100g)** | | |  | |  | | | **Sugar/fat content (g/100g)** | |
|  | **Sugar** | | **Fat** |  | **Food (total=140)** | | | | **Fat** | **Sugar** |
|  |  | |  |  |  | | | |  |  |
| Almonds | 5.7 | | 53.0 |  | Dark chocolate praline | | | | 41.6 | 37.0 |
| Almond flavored popsicle | 29.0 | | 21.0 |  | Dates | | | 64.0 | | 0.5 |
| Amarettini | 76.2 | | 10.1 |  | Deep-fried anchovies | | | 1.0 | | 22.0 |
| Apricots | 7.7 | | 0.1 |  | Donut with chocolate frosting | | | | 20.0 | 33.3 |
| Asian noodles | 3.0 | | 5.0 |  | Double choc cookie | | | | 44.0 | 23.0 |
| Avocado | 0.7 | | 12.0 |  | Dried plums | | | | 28.1 | 0.4 |
| Bacon Chips | 3.3 | | 21.0 |  | Drop cake | | | | 20.0 | 3.5 |
| B­­aklava | 61.0 | | 8.4 |  | Drop cake with chocolate frosting | | | | 20.0 | 3.5 |
| Bean stew | 1.5 | | 2.2 |  | Egg waffles | | | | 33.0 | 26.0 |
| Bell pepper | 5.0 | | 0.3 |  | Flan caramell | | | | 6.0 | 3.0 |
| Black licorice wheels | 47.0 | | 0.1 |  | Fried mushrooms | | | | 0.0 | 0.3 |
| Black olives | 0.0 | | 13.2 |  | Fries | | | | 0.0 | 12.0 |
| Blackberries | 2.7 | | 1.0 |  | Frog shaped wine gum | | | | 53.2 | 0.1 |
| Blue grapes | 15.4 | | 0.3 |  | Fruit bar | | | | 50.0 | 5.0 |
| Blueberries | 6.0 | | 0.6 |  | Fruit gum stripes | | | | 54.0 | 0.9 |
| Blueberry muffin | 22.0 | | 22.0 |  | Galia melon | | | | 3.6 | 0.0 |
| Brawn | 0.0 | | 17.6 |  | Ginger bread | | | | 35.0 | 15.8 |
| Broccoli | 2.7 | | 0.2 |  | Goulash | | | | 0.0 | 3.0 |
| Buttered bread | 1.6 | | 26.8 |  | Grapefruit | | | | 6.9 | 0.2 |
| Candy bar with caramel | 61.7 | | 16.6 |  | Green beans | | | | 1.2 | 0.2 |
| Caramel soft candy bar | 35.6 | | 37.5 |  | Green bell pepper | | | | 2.8 | 0.2 |
| Cashew nuts | 9.1 | | 42.0 |  | Green olives | | | | 0.0 | 17.4 |
| Cauliflower | 1.9 | | 0.3 |  | Hazelnuts | | | | 4.6 | 62.0 |
| Cellery stalks | 1.8 | | 0.2 |  | Hazelnut flavored chocolate bar | | | | 50.9 | 33.1 |
| Chestnuts | 8.0 | | 0.4 |  | Herring | | | | 0.0 | 15.0 |
| Chocolate-coated cornflakes | 5.7 | | 28.0 |  | Honeydew melon | | | | 5.3 | 0.1 |
| Chocolate covered fruits | 41.6 | | 13.8 |  | Khaki | | | | 16.0 | 0.3 |
| Chocolate cream tartlet | 40.0 | | 28.0 |  | Köttbullar | | | | 0.6 | 20.0 |
| Chocolate filled popsicle | 22.0 | | 21.0 |  | Mandarin orange | | | | 10.0 | 0.3 |
| Chocolate lentils | 53.5 | | 25.5 |  | Maracuya | | | | 9.5 | 0.4 |
| C­­­hocolate marshmallow | 53.0 | | 9.7 |  | Meatloaf | | | | 0.0 | 26.0 |
| Chocolate nut waffles | 42.6 | | 31.9 |  | Meat stew with vegetables | | | | 1.5 | 2.7 |
| Clams | 0.0 | | 3.1 |  | Milk cream layered candy bar | | | | 29.5 | 27.9 |
| Coconut chocolate bar | 48.2 | | 25.7 |  | Mixture of wine gums | | | | 55.0 | 2.2 |
| Corn | 5.2 | | 1.9 |  | Mueslibar | | | | 0.0 | 11.3 |
| Couscous salad | 19.0 | | 9.8 |  | Nashi peach | | | | 7.0 | 0.2 |
| Crisp bread | 7.5 | | 9.0 |  | Nectarine | | | | 12.3 | 0.1 |
| Crispy nut biscuit | 31.0 | | 35.3 |  | Nuts in chocolate | | 47.0 | | | 35.1 |
| Croquettes | 1.0 | | 8.0 |  | Nut chocolate praline | | | | 39.9 | 42.7 |
| Currant bun | 21.0 | | 9.3 |  | Octopus salad | | | | 0.0 | 10.0 |
| Dark chocolate | 47.8 | | 30.1 |  | Olives with bell pepper | | | | 0.0 | 15.0 |
| **Supplementary Table S1: continued** | |  |  |  |  | | | |  |  |
|  |  | |  |  |  | | | |  |  |
| **Food (total=140)** | **Sugar/Fat (g/100g)** | | |  | |  | | | **Sugar/fat content (g/100g)** | |
|  | **Sugar** | | **Fat** |  | **Food (total=140)** | | | | **Sugar** | **Fat** |
|  |  | |  |  |  | | | |  |  |
| Orange | 8.2 | | 0.2 |  | Toast with jam | | | | 14.3 | 9.9 |
| Peach | 10.3 | | 0.3 |  | Tofu salad | | | | 9.0 | 23.0 |
| Peas and carrots | 4.6 | | 2.3 |  | Tomatoes | | | | 2.5 | 0.2 |
| Pickled garlic | 1.9 | | 2.6 |  | Tortellini | | | | 1.9 | 2.0 |
| Pickled tomatoes | 6.3 | | 29.6 |  | Vanilla pudding | | | | 11.0 | 8.2 |
| Pineapple | 12.2 | | 0.2 |  | Vegetables with Asian noodles | | | | 3.5 | 1.5 |
| Pistachios | 5.8 | | 50.0 |  | White chocolate-coated fruit | | | | 78.0 | 32.1 |
| Pitahaya | 7.0 | | 0.4 |  | White chocolate-coated cornflakes | | | | 38.5 | 28.3 |
| Pizza | 4.0 | | 7.1 |  | White mushrooms | | | | 0.2 | 0.2 |
| Pizza bread | 9.3 | | 22.0 |  | Wiener | | | | 0.0 | 23.0 |
| Polish sausage | 0.3 | | 8.0 |  | Wine gum | | | | 46.0 | 0.4 |
| Popcorn | 27.9 | | 26.3 |  | Yellow apple | | | | 10.4 | 0.2 |
| Popsicle | 18.0 | | 0.5 |  | Yellow jelly | | | | 17.4 | 0.0 |
| Praline | 54.5 | | 33.0 |  | Zucchini | | | | 1.8 | 0.4 |
| Pretzel pieces | 6.5 | | 28.0 |  |  | | | |  |  |
| Pretzelbreadstick | 0.5 | | 4.6 |  |  | | | |  |  |
| Raclette cheese | 0.0 | | 25.0 |  |  | | | |  |  |
| Raw Brussel sprouts | 2.8 | | 0.3 |  | Source: FDDB, “Food Data Base”, [Online]. | | | | | |
| Red apple | 10.0 | | 0.6 |  | Available: http://fddb.info | | | | | |
| Red bell pepper | 6.4 | | 0.5 |  |  |  |  |  |  |  |
| Red grapes | 15.4 | | 0.3 |  |  |  |  |  |  |  |
| Red pointed pepper | 6.4 | | 0.5 |  |  |  |  |  |  |  |
| Roasted almonds | 41.0 | | 38.0 |  |  |  |  |  |  |  |
| Rocket salad | 1.7 | | 0.7 |  |  |  |  |  |  |  |
| Salami | 0.5 | | 32.0 |  |  |  |  |  |  |  |
| Salt sticks with sesame | 2.80 | | 8.7 |  |  |  |  |  |  |  |
| Saltine crackers | 6.8 | | 19.0 |  |  |  |  |  |  |  |
| Sandwich | 3.8 | | 16.4 |  |  |  |  |  |  |  |
| Sauerkraut with bacon | 1.3 | | 2.2 |  |  |  |  |  |  |  |
| Sausage salad | 4.8 | | 22.0 |  |  |  |  |  |  |  |
| Scrambled egg | 1.0 | | 4.6 |  |  |  |  |  |  |  |
| Semolina pudding | 44.0 | | 10.0 |  |  |  |  |  |  |  |
| Short bread biskuits | 23.5 | | 26.9 |  |  |  |  |  |  |  |
| Soft cheese | 0.5 | | 33.0 |  |  |  |  |  |  |  |
| Strawberries | 5.3 | | 0.4 |  |  |  |  |  |  |  |
| Strawberry waffle ice cream | 29.0 | | 11.0 |  |  |  |  |  |  |  |
| Stuffed cherry-pepper | 1.0 | | 9.8 |  |  |  |  |  |  |  |
| Stuffed mushrooms | 1.0 | | 10.9 |  |  |  |  |  |  |  |
| Surimi | 2.7 | | 5.8 |  |  |  |  |  |  |  |
| Tabouleh salad | 5.1 | | 1.8 |  |  |  |  |  |  |  |
| Tilsit | 0.1 | | 28.0 |  |  |  |  |  |  |  |

**Supplementary Table S2: Multiple regression results**

| **Criterion** | **Model (R²)** | ***Predictor variables (standardized β – coefficients)*** | | | | |
| --- | --- | --- | --- | --- | --- | --- |
|  |  | VTA-NAc | NAc-LH | BMI | BMI x VTA-NAc | BMI x NAc-LH |
| *Sugar wanting* |  |  |  |  |  |  |
| Reg.1 | .340*** | .166 |  | -.193 | .558*** |  |
| Reg.2 | .041 |  | .068 | -.184 |  | -.022 |
| Reg.3 | .381** | .124 | .053 | -.195 | .620*** | -.201 |
| *Fat wanting* |  |  |  |  |  |  |
| Reg.1 | .126 | .032 |  | -.201 | .302 |  |
| Reg.2 | .079 |  | -.063 | -.197 |  | .182 |
| Reg.3 | .143 | .080 | -.082 | -.201 | .268 | .106 |

**p < 0.01 ***p < 0.001.

**Supplementary Table S3: Sample Characteristics**

|  | **All (N = 45)** | **BMI_norm_ (N = 23)** | **BMI_over_ (N = 22)** | **p** |
| --- | --- | --- | --- | --- |
| Age | 25.8 (3.2) | 25.9 (3.5) | 25.7 (2.9) | NS |
| Sex (female/male) | 22/23 | 11/12 | 11/11 | NS |
| BMI (kg/m²) | 25.5 (4.7) | 21.5 (1.8) | 29.6 (2.9) | *** |
| Time fasted (hours) | 13.0 (1.4) | 12.7 (1.5) | 13.3 (1.3) | NS |
| Hunger rating (1 – 10) | 4.3 (2.5) | 4.4 (2.3) | 4.2 (2.7) | NS |
| Glucose (mmol/L) | 4.8 (0.4) | 4.7 (0.3) | 4.9 (0.4) | NS |
| HbA1C (%) | 4.9 (0.3) | 4.9 (0.3) | 4.9 (0.3) | NS |

BMI, body mass index; NS, not significant; *** p < 0.001, with standard deviation in brackets


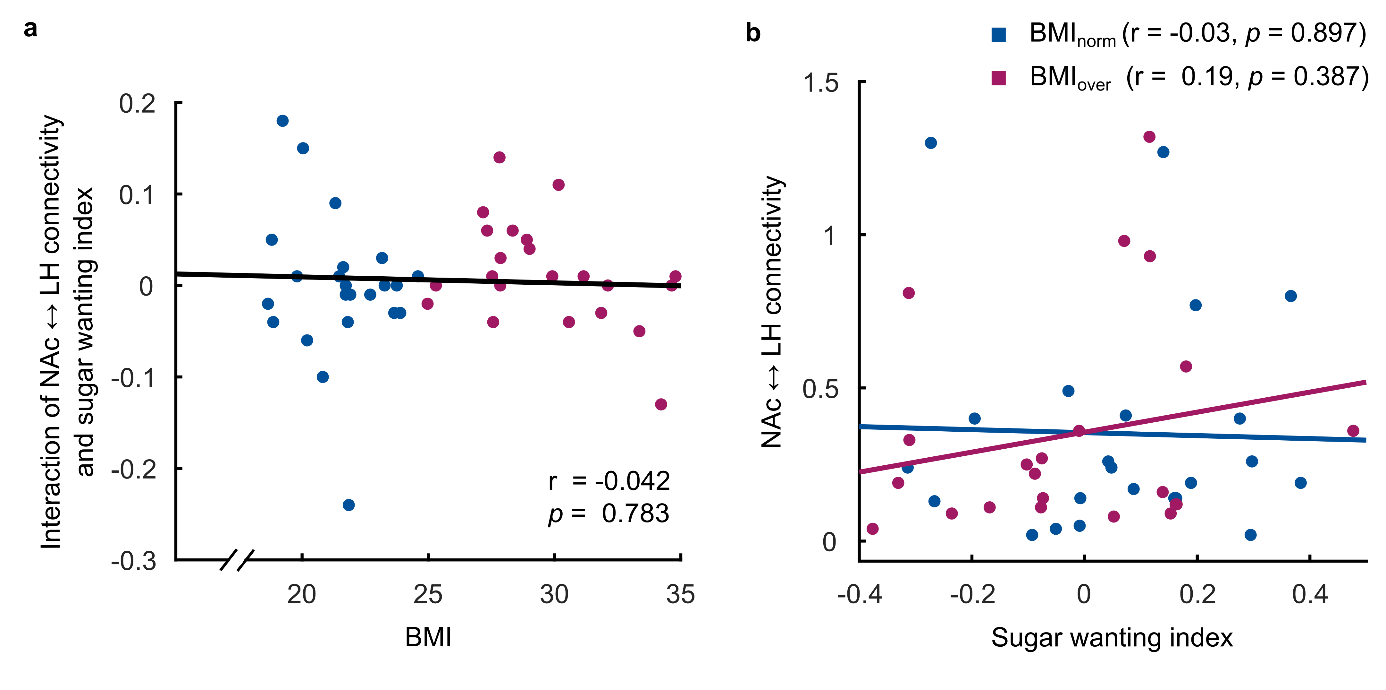


**Supplementary Figure S1. Interaction of NAc-LH and sugar wanting and its relationship with BMI. (a)** The interaction of sugar wanting index and NAc-LH connectivity did not correlate significantly with BMI. **(b)** Accordingly, there was no group-wise correlation between NAc-LH connectivity and the sugar wanting index.

**Supplementary results**

To explore potential differences between tracts as well as BMI interactions we computed an ANOVA including VTA-NAc and NAc-LH connectivity indices and BMI group factor. Results demonstrate a main effect of tracts, i.e. stronger connectivity between VTA-NAc compared to NAc-LH (F(1,43) = 13.47, P < .001) but no group interaction (F(1,43) = 1.24, P = 0.272).
